# Smart Resource Allocation in Mobile Cloud Next-Generation Network (NGN) Orchestration with Context-Aware Data and Machine Learning for the Cost Optimization of Microservice Applications

**DOI:** 10.3390/s24030865

**Published:** 2024-01-29

**Authors:** Mahmood Ul Hassan, Amin A. Al-Awady, Abid Ali, Muhammad Munwar Iqbal, Muhammad Akram, Harun Jamil

**Affiliations:** 1 Department of Computer Skills, Deanship of Preparatory Year, Najran University, Najran 66241, Saudi Arabia; mahmood.mscs@gmail.com (M.U.H.); aaalawady@nu.edu.sa (A.A.A.-A.); 2 Department of Computer Science, University of Engineering and Technology, Taxila 48080, Pakistan; munwariq@gmail.com; 3 Department of Computer Science, Govt. A.N.K. (S) Degree College K.T.S., Haripur 22620, Pakistan; 4 Department of Computer Science, College of Computer Science and Information Systems, Najran University, Najran 66241, Saudi Arabia; akram.moghal@gmail.com; 5 Department of Electronic Engineering, Jeju National University, Jeju-si 63243, Republic of Korea; faiharunjamil@jeju.nt.kr

**Keywords:** machine learning, mobile cloud computing, power consumption, cost estimation, task sequencing, task scheduling, microservices

## Abstract

Mobile cloud computing (MCC) provides resources to users to handle smart mobile applications. In MCC, task scheduling is the solution for mobile users’ context-aware computation resource-rich applications. Most existing approaches have achieved a moderate service reliability rate due to a lack of instance-centric resource estimations and task offloading, a statistical NP-hard problem. The current intelligent scheduling process cannot address NP-hard problems due to traditional task offloading approaches. To address this problem, the authors design an efficient context-aware service offloading approach based on instance-centric measurements. The revised machine learning model/algorithm employs task adaptation to make decisions regarding task offloading. The proposed MCVS scheduling algorithm predicts the usage rates of individual microservices for a practical task scheduling scheme, considering mobile device time, cost, network, location, and central processing unit (CPU) power to train data. One notable feature of the microservice software architecture is its capacity to facilitate the scalability, flexibility, and independent deployment of individual components. A series of simulation results show the efficiency of the proposed technique based on offloading, CPU usage, and execution time metrics. The experimental results efficiently show the learning rate in training and testing in comparison with existing approaches, showing efficient training and task offloading phases. The proposed system has lower costs and uses less energy to offload microservices in MCC. Graphical results are presented to define the effectiveness of the proposed model. For a service arrival rate of 80%, the proposed model achieves an average 4.5% service offloading rate and 0.18% CPU usage rate compared with state-of-the-art approaches. The proposed method demonstrates efficiency in terms of cost and energy savings for microservice offloading in mobile cloud computing (MCC).

## 1. Introduction

Mobile application development is gaining importance due to mobile devices’ user-friendly and portable features. The applications running on mobile devices provide services through the internet using mobile cloud computing (MCC) integration [1]. The quality of mobile applications depends on the services that they deliver. The key fundamental elements of such applications are quality of service (QoS), time consumption, application running cost, and power consumption, which are very intense and impactful. In our assessment, we consider these characteristics optimal for users. Quality of experience (QoE) is another feature that measures the satisfaction provided to users while they use such applications [2]. The QoE parameter is linked to the longest possible uptime of mobile devices, with this parameter being used to ensure less power and minimum resource cost consumption.

Moreover, the QoE parameter shows an application’s performance, which provides user satisfaction by ensuring the delivery of non-stop services to mobile users [3]. Tasks are considered microservice-based and provide both dependent and independent services in each mobile application. Microservices provide a more accurate delivery of QoS and QoE in MCC. Cloud computing (CC) has the added benefits of ubiquitous networks, on-demand services, and resource pooling for mobile users. MCC implements Wi-Fi, 4G, and 5G capabilities to facilitate the processing and execution of activities. It facilitates the organization of task offloading in mobile cloud computing and the determination of whether tasks should be processed on the mobile device or via mobile cloud orchestration [4].

Besides all these options, the power consumption of a mobile device provides the quality parameters that ensure the smooth running of mobile applications. These quality parameters of mobile applications and devices ensure operation at both hardware and software levels. Quality parameters are cost and power consumption, which improve task scheduling through machine learning [5]. The task scheduling processing of information allows mobile applications to improve quality parameters and processing power. Some recent research articles describe the machine learning solution in terms of its limitations, i.e., specific application scenarios for mobile devices and offline model adaptation for the system architecture [6]. Ultimately, the power consumed by the mobile devices is not in agreement with the provided case scenarios and enhanced the learning experience. A generic solution is adopted using these previous techniques, enabling the working of only a few of the solutions for all mobile applications. Dynamic adaptation and a fully optimized solution are presented. This research promotes effective resolution and enhances coordination control for online mobile applications. All microservice-based applications are executed on mobile devices.

Cost and time are the main parameters for mobile and other service-oriented solutions that help reduce mobile devices’ power consumption and cost [7]. In distributed systems, microservice design is crucial, as it significantly lower expenses. The microservice architecture provides the flexibility to scale services, isolate faults, and deploy services independently. This is accomplished by decomposing a complex application into smaller, loosely coupled services, each accountable for a distinct purpose. This modular approach encourages quicker development and testing cycles by allowing teams to work on separate services concurrently. Microservices may also be scaled separately, which optimizes resource distribution and lowers infrastructure costs. The fault isolation attribute also ensures that a service failure does not affect the entire system, increasing fault tolerance and resilience. In conclusion, the microservice architecture is a crucial strategy for distributed systems because of its capacity to lower costs through scalability, fault isolation, and independent deployment [8].

Reinforcement learning is a classical approach for online learning platforms. We present our problem’s supervised learning solution, executed in both online and offline contexts. In this approach, just like reinforcement learning, the training data and trained model can be automatically extended and updated in any stage. The research problem that the authors address in this article relates to the practical problem that different societies face while learning through mobile cloud computing [9]. The proposed approach makes knowledge discovery possible, even when other cloud services are not specifically present. This research also resolves the privacy and scalability issues encountered when mobile devices operate offline. The next section provides the motivation for the study.

Mobile devices often have limited resources regarding battery energy, a low CPU clock speed, and inadequate sensing capacities and storage space. MCC offloads resource-hungry/resource-intensive tasks. Context-aware services are prevalent among end-users due to their relevance and better QoE. MCCs are expected to adapt seamlessly to vigorous changes in end-user contextual information. The delay should be minimal in context-aware services to increase task efficiency. In addition, this article aims to reduce latency for optimizing tasks. Machine learning and artificial intelligence effectively accomplish high precision in mobile computational issues. The training and testing outcomes are linked to machine applications that prioritize the needs and preferences of the user. Microservices-based applications handle mobility, power, and cost as challenges in the MCC paradigm. A context-aware ML-based model is required to offload tasks with minimum cost and energy consumption [10].

This paper uses machine learning to examine the energy- and cost-efficient task scheduling problem in mobile cloud computing applications. The authors consider the VM-based context-aware mobile cloud computing network. The research goal is to save power and consume less cost for microservices-based mobile applications. The associated vector attributes include tasks, CPU instructions, data size, and execution deadlines. The authors consider MCC services based on their cost and energy. The authors consider the questions below during task scheduling in this paper.

How do we consider a context-aware energy- and time-efficient MCC model for task scheduling using an enhanced microservices-based approach?

- How can the appropriate machine learning model be selected to effectively train and test microservices-based task scheduling?
- How can cost and energy efficiency be achieved through the proposed solution?
- How can optimal task scheduling be performed to reduce operating costs and task execution under defined deadlines?
- This research contributes to the cost and energy efficiency of microservices-based mobile applications using MCC and machine learning.

Context-aware task offloading for mobile devices saves mobile application resources. The authors propose a novel microservices-based context-aware machine learning model. The authors implement a supervised machine learning model to improve cost and energy efficiency and enhance system performance. The proposed approach uses less cost and energy to offload tasks to MCC.

This study uses novel context-aware microservices-based tasks to offload using reinforcement learning-based approaches. The customized novel task scheduling algorithm offloads tasks using cost-effective and short timeframes compared to previous techniques. The proposed methodology uses fault tolerance and resource-constrained tasks to consume less time and offloading cost.

This paper considers server-based optimized task scheduling policies for task offloading and decision making. The authors consider QoS for each individual microservice to implement the offloading policies for microservices-based applications. A service matching algorithm is implemented using machine learning features such as services and tasks. This is carried out to compare and execute instructions based on service requirements.

Graphical outcomes are shown to ascertain the efficacy of the intended endeavor. At a service arrival rate of 80%, the suggested model obtains an average service offloading rate of 4.5% and a CPU consumption rate of 0.18% compared to state-of-the-art techniques. The suggested solution exhibits cost and energy conservation efficacy for microservices offloading in mobile cloud computing (MCC).

The processing module obtains the tasks from mobile devices and flow system and develops the proposed technique for task scheduling. All tasks are handled sequentially to provide and enhance task development. The authors send microservices-based tasks from mobile devices to machine learning algorithms. Once the model is trained and tested, the remaining tasks are handled through the proposed trained model.

The tasks become challenging in terms of handling the computational cost and optimizing energy. To address these problems, the authors propose a microservices-based energy and cost-efficient machine-learning-based task scheduler.

The remainder of this article is structured as follows: Section 2 presents a literature review of the contributions of state-of-the-art-related academic research on task scheduling using different machine learning algorithms. Then, in Section 3, the authors design a machine-learning-based novel task scheduling approach for the implementation of modern microservices context-aware tasks beyond the capacity of mobile devices, which perform all the required operations on mobile devices. This section describes the complete implementation in detail, in addition to the justified parameters and algorithms used to implement the proposed methodology. The proposed algorithm works on context-aware microservices-based tasks using reinforcement learning, while existing techniques only use normal methods with average results, as mentioned in Table 1’s literature section. Section 4 presents a practical implementation using a simulation of the machine learning model and MCC network. A dataset is used for tasks created with a random length between 5000 and 8000 MIPS. In each set, the number of tasks is 500–750. The parameters of the dataset include the total length of each task, number of tasks, number of VMs, average load, task type, the CPU time required, difference time in average arrivals, CPU utilizations, bandwidth utilization, the task failure ratio, and task boot time. The accuracy is 97% with a 4.5% better service offloading rate and 0.18% lower CPU usage than existing state-of-the-art approaches. Section 5 shows the conclusion and future direction for related research with the fault tolerance technique.

## 2. Literature Review

**Figure 1:**
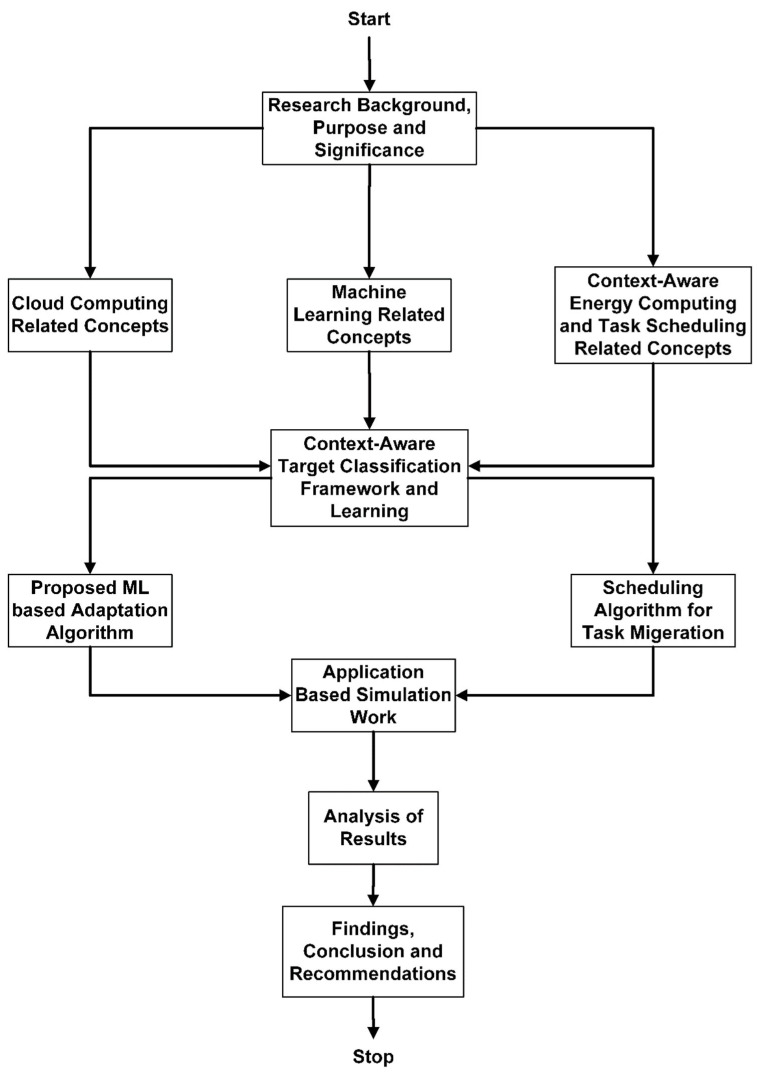
Workflow of proposed system with implementation.

Power management issues are becoming one of the most demanding research issues in mobile cloud computing (MCC). Virtualization and cloud computing technologies work together to keep a business up to date for correspondence and context-aware scheduling. VMs are assigned to every task migrated to the cloud VM. The current system effectively migrates the tasks that machine learning predicts in Algorithm 1. All tasks are considered critical in this methodology. Only the tasks with the highest computation time are not migrated to VMs. The tasks’ final completion time is computed using these tasks in VMs. A task scheduling algorithm is used to determine the computation values of the task. Context-aware tasks with the highest priority assigned during task optimization are scheduled and subjected to the task’s optimization technique [11]. Figure 1 shows the workflow of the proposed system with implementation details.
**Algorithm 1:** Suboptimal Decision Making**Input:** Tasks, Machine Learning Predictions, Number of VMs,          Speed, Location, Time, Energy and Cost. **Output:** Train and Test the Model for Sub−Optimal Decision Making **Steps** Get_Tast() Get inf0 (L, T, E, C, S, T) $if\left(K\neq \emptyset \right)\;then$            train_model(K,P,T,A,C,L); $while\left(d\in D\&\&r_{i}\in R_{es}\right)\;do$ $Predict_{Results\left(\right)}\leftarrow r_{i}\left(d,x\right)$ end if          R=store(Training Results)            ${execute}_{OCR}\left({MCC}_{VM}\right)$           $if(T_{success}<Traing)$      $test\_model(random_{position},\;energy,\;time-predict)$ end if        $R_{visualize}\leftarrow Traing,\;Test,\;Validate,\;compare$ Go to Step 1

Power management issues are the most relevant in mobile cloud computing, especially task scheduling. In [12], the author proposed a modern distributed system with cloud services and virtualization features. The authors proposed a new power management technique using machine learning for task scheduling in MCC. The approach automates the power of different systems to distribute their features. Although research has contributed to task scheduling, there is a lack of explanation of machine learning algorithms, and they are not adapted to relevant contexts. In this approach, the authors only consider desktops and similar systems. Still, they do not focus on mobile devices, which are the core of the MCC technique and an essential part of modern mobile-equipped distributed systems. Power management became important after the introduction of mobile devices in mobile cloud computing. Various studies have been conducted in this regard, including [13,14].

In some cases, authors focused on generalizing and discussing power management in MCC with and without machine learning [14]. Alongside power management, energy-saving strategies for mobile device applications are major focus areas for task scheduling and submission in mobile cloud computing [15]. The author in [16] demonstrated the energy efficiency of mobile devices at the software and hardware levels.

Additionally, the author discussed power management at the operating system level. Sensors, interfaces, and other devices for cloud computing are also discussed in [17]. Sequential programs, mobile computing offloading, and GUI-based design are also considered for task scheduling in mobile cloud computing [18]. In addition, some strategies are implemented for task scheduling to save the battery power of mobile devices using mobile cloud computing [19]. The authors compute the offloading calculation time for power saving on mobile devices through the proposed mathematical model. Power saving and other related aspects are distributed through MCC. Besides its contributions, this research uses more CPU from a data security point of view, which causes the system to consume more power than usual due to data encryption processes. Another reason for this is the communication overhead, which causes tasks to consume more power from mobile devices. This research does not address practical considerations. It only discusses the theoretical consideration of power saving with a machine learning algorithm.

In [20], the author conducts similar research in mobile application offloading through cloud computing to increase efficiency and balance power consumption. This technique uses cloudlet-based task offloading rather than MCC. The idea is to offload the application to resource-rich devices. This research considers cloudlet and mobile device load availability for applications, but it lacks simulation-based experiments. Theoretical tests are conducted to develop practical application scenarios. Another technique in [21] uses the cloudlet-based approach for multilevel full and partial task offloading. A test is developed using real solution-based devices. Another technique in [22] uses trustable full task offloading in mobile cloud computing. This approach develops a simulation-based test environment to offload the tasks to MCC.

Reference [23] is another article based on mobile application power consumption using mobile devices for e-learning. The researchers developed a mobile application that performs two tasks simultaneously: a task that considers power consumption and a task that considers the cost to offload a task to a cloud application. The authors critically analyzed the proposed system by comparing it with existing modeling, power management, and adaptation techniques. This research lacks an analysis of machine learning implemented to run applications on mobile devices in order to examine mobile devices’ power consumption. The communication aspect of mobile devices also contributes significantly to processing power.

Energy optimization is achieved through machine learning, which is a popular energy optimization technique. In [24], the authors propose it in large-scale systems like data centers for task scheduling. The paper suggests that machine learning predicts system parameters like CPU load, power consumption, and cost. Although this is like ours, this approach is not applied to mobile devices. Another downside is that this technique only performs a single task for validation and performs different analyses and learning tasks through offline processes. The article in [25] comprehensively reviews various cost optimization techniques in mobile cloud computing. It highlights the importance of context awareness and machine learning for effective resource allocation. The study emphasizes the significance of considering network conditions, user context, and application demands to allocate resources efficiently in microservices applications. The article in [26] proposes a context-aware resource allocation model for microservices in MCC. The model considers user context, device capabilities, and application requirements to allocate resources dynamically. The study demonstrates the effectiveness of the proposed approach in optimizing costs and improving application performance, particularly in scenarios with varying network conditions. The paper adopts reinforcement learning for cost optimization in microservices applications deployed in MCC and under changing conditions and user requirements. The study demonstrates the potential of reinforcement learning in achieving cost-effective resource utilization while maintaining service quality in dynamic mobile scenarios. This research investigates energy-aware task offloading and resource allocation in mobile edge computing for microservices applications. The study integrates context-awareness into decision making to optimize energy consumption and overall costs. The suggested method decreases the time delay and improves the efficiency of applications. Edge computing is adopted to improve resource efficiency.

So far, all studies in the literature have conducted machine-learning-based cost and power analyses. The context-aware features in these frameworks perform different MCC task analyses. In [26], the authors use machine-learning-based context-aware scheduling for task processing. The presented technique addresses and enables the network parameters’ dynamic adaptation. There is no user interaction for selecting the parameters for different scenarios. The authors design an offloading algorithm to conduct a proper analysis. The mobile device’s behavior is predicted and sufficiently detailed to analyze the context. The authors analyze the proposed technique using four machine learning algorithms, i.e., Linear Logistic Regression, Linear Discernment Analysis, Support Vector Machine, and K-Nearest [27]. The authors consider this in the mobile cloud computing framework. The system uses Android OS to accept mobile tasks and offload tasks properly. The proposed system determines the average power consumption of the mobile states. The power consumption of PowerTutor, GPS, and other related interfaces is measured. The actual context is available for the mobile devices to carry out the test that effectively utilizes the proposed technique. Although the technique is effectively adopted for determining the power consumption of the test module of mobile devices, in real-world scenarios, this application is limited in its capabilities [28]. The existing algorithms used for task scheduling and optimization include the Prairie Dog Optimization Algorithm [29], Dwarf Mongoose Optimization Algorithm [30], Aquila Optimizer [31], Reptile Search Algorithm [32], Ebola Optimization Search Algorithm [33], and Arithmetic Optimization Algorithm [34].

Moreover, the cost efficiency of mobile devices is limited with limited features and capabilities. The authors believe that power consumption and energy optimization for approaches in real-world scenarios are limited to energy efficiency and the cost estimation of offloading tasks to cloud machines. The authors focus on cost efficiency and power management in context-aware task scheduling, and they highlight the problems that motivated them to continue the research in this field. Based on our proposed architecture and in support of [35], we propose a genetic-based clustering algorithm to optimize resource allocation in IoT applications deployed on layered fog-based heterogeneous platforms. The study addresses the challenges of limited resources and heterogeneity within fog computing environments by proposing a novel clustering approach. The algorithm utilizes a genetic-based technique to allocate resources efficiently, considering factors such as application requirements and fog node capabilities. Using simulation experiments and performance evaluations, the authors demonstrate this approach’s effectiveness in improving resource allocation, reducing latency, and enhancing overall system performance. The research contributes to the field by offering a promising solution for resource management in microservices-based context-aware applications deployed in fog computing environments, paving the way for enhanced scalability and efficiency. Our approach focuses on task scheduling for microservices-based optimization in machine learning techniques. Table 1 compares related work based on the characteristics of the proposed method. Based on Table 1, the hypotheses/baselines are as follows:

**Hypothesis** **1.** *Implement task scheduling based on machine learning and time, fault rate, and MCC VM server utilization.*

**Hypothesis** **2.** *Schedule tasks based on MCC VM server utilization, latency values, sequencing, boot time, CPU utilization, and time.*

**Hypothesis** **3.** *Implement task scheduling based on machine learning (ML), MCC server utilization, fault tolerance, task boot time, and task schedule latency.*

**Hypothesis** **4.** *Implement task scheduling based on MCC server utilization, fault tolerance, task deadline completion, cost efficiency, resource handling, and task schedule latency.*

**Hypothesis** **5.** *Implement task scheduling based on MCC server utilization.*

**Hypothesis** **6.** *Implement task scheduling based on ML, task deadline completion, and resource handling.*

**Hypothesis** **7.** *Implement task Scheduling based on ML, fault tolerance, task boot time, and resource handling.*

**Hypothesis** **8.** *Implement task scheduling based on fault tolerance and task schedule latency.*

**Hypothesis** **9.** *Implement task scheduling based on fault tolerance, task deadline completion, and cost efficiency.*

**Hypothesis** **10.** *Implement task scheduling based on MCC server utilization, task boot time, and resource handling.*

**Hypothesis** **11.** *Implement task scheduling based on machine learning, MCC server utilization, fault tolerance, task deadline completion, task boot time, cost efficiency, resource handling, and Task schedule latency.*

This research is a continuation of our published research articles in [5,6]. In [6], the authors utilize task scheduling for MCC using a customized algorithm to effectively enhance the proposed scenario’s processing capabilities. In [5], the authors focus on security-aware task scheduling using a multilevel trust management approach to schedule optimization techniques effectively. In [19], the authors utilize different optimization techniques for task scheduling and energy management in the proposed method. Using these techniques, simulation-based tests are conducted to obtain research results for practical analyses and procedures. The authors use microservices-based task offloading in this proposed approach for context-aware applications. The authors use systematic techniques to focus on the cost efficiency and power management of all mobile devices.

Previous studies handle task scheduling for microservices-based applications by using a traditional statistical and computational approach, which cannot be handled by many modern mobile applications. Cost efficiency and processing power are not handled effectively in task scheduling, as mentioned in Table 1. This paper aims to reduce the computational power, energy consumption, and communication costs of MCC and mobile devices. In the next section of this paper, the authors explain the details of the proposed architecture. In Table 1, “-” indicates that the methodology does not consider the mentioned parameter in the column heading, and “✓” shows that the mentioned paper considers the parameter in their experiments.

**Table 1:** Comparison of related work based on characteristics of proposed system.

Papers	Hypothesis/Baseline	Machine Learning	MCC Server Utilization	Fault Tolerance	Task Deadline Completion	Task Boot Time	Cost Efficiency	Resource Handling	Task Schedule Latency	Throughput	Network Latency
[22]	H1	-	✓	-	✓	✓	✓	-	-	-	-
[23]	H2	-	✓	-	-	✓	-	-	✓	-	-
[24]	H3	✓	✓	✓	-	✓	-	-	✓	-	-
[27]	H4	-	✓	✓	✓	-	✓	✓	✓	-	-
[28]	H5	-	✓	-	-	-	-	-	-	-	-
[30]	H6	✓	-	-	✓	-	-	✓	-	-	-
[31]	H7	✓	-	✓	-	✓	-	✓	-	-	-
[32]	H8	-	-	✓	-	-	-	-	✓	-	-
[33]	H9	-	-	✓	✓	-	-	-	✓	-	-
[34]	H10	-	-	✓	✓	-	✓	-	-	-	-
[35]	H11	-	✓	-	-	✓	-	✓	-	-	-
Proposed Methodology	H12	✓	✓	✓	✓	✓	✓	✓	✓	-	-

## 3. Supervised Machine Learning Techniques for Microservices-Based Mobile Devices

This section examines supervised learning using an approximation function, wherein items in set X are labeled from set C. The emphasis is on discovery, which involves extracting knowledge from the function’s values and arguments. This process benefits supervised machine learning algorithms by facilitating efficient information flow, and it presents a feature set, P, and an alternative representation, $x^{P}$. This allows for an investigation of the classification-dependent size of set C, a discussion of the classification-dependent size of set C, and a discussion of the hypotheses as the fundamental components for generating knowledge when using different machine learning techniques. These techniques include support for Bayesian Networks and Naïve Bayes. The approximation function f:X→C is used in supervised learning. The labels are assigned from the C set to X objects for every microservice in mobile applications. Knowledge discovery is required to generate knowledge from f values and their arguments. Labeling is beneficial for supervised machine learning algorithms for the efficient flow of information. The authors assume that feature set $P=(p_{1},p_{2},p_{3},\ldots \ldots p_{n})$, where $p_{i}:X\to Q_{i}.$ Therefore, $x^{P}=(p_{1}\left(x\right),p_{2}\left(x\right),p_{3}\left(x\right),\ldots \ldots \ldots ,p_{n}\left(x\right))$ is used as an alternative to x. The size of C is dependent on classification or non-classification. If the size is small, then learning for classification is required. C always comprises the set of classes required for classification and is called the k classifier. As the authors know, the supervised ML tool obtains trained data containing this set. $\left\{x^{P}f(x)\right\}$ Hypothesis h contains general knowledge about the target ML technique. In this context, the problem would be $x^{P}$, and its answer would be $h\left(x^{P}\right)$. Various ML methods are adopted for supervised learning to enhance knowledge about astringent hypotheses. The hypothesis is the main building block in generating knowledge from numerous techniques, eliminating the proposed processes. The main methods used to generate hypotheses are discussed below.

To support the Bayesian Network (BN), Naïve Bayes (NB) is used as a probabilistic classifier for profound learning decisions. The BN contains a set of two pairs, i.e., (G, P). G is the graphical structure, and P is the probability distribution of local conditions among the features used in the network and their parents. G is considered a simple structure graph. C is the class node and is regarded as the parent of every attribute node. $p_{1},p_{2},p_{3},\ldots \ldots p_{n}$. Figure 1 shows the learning agent that adopts mobile cloud energy optimization for task offloading systems.

In supervised ML, learning is considered a conditional probability $P\left(\left\{p_{i}|C\right\}\right)$ and a priori probability P(C). The mobile device task probability distribution is computed through Equation (1):

(1) $$P\left(C\left(x\right)=C_{k}\right)\leftarrow \frac{P\left(C_{k}\right)\prod_{i=1}^{n}P(p_{i}\left\{x|C_{j}\right\})}{\prod_{i=1}^{n}P(p_{i}(x))}$$

In Equation (1), the probability distribution function is computed through $p_{1}\left(x\right),p_{2}\left(x\right),p_{3}\left(x\right),\ldots \ldots \ldots ,p_{n}\left(x\right)$. The C4.5 learning algorithm is used to learn the system. C4.5 is based on a decision tree. A decision tree learns through examples recursively. Training is based on the same class labels. The same class tree is returned after labeling the leaf node of the tree. For practical training through entropy measurement, the best attribute is the root. Attributes represent the training values of the model, and their values are trained recursively. Examples are used as parameters for testing the values with appropriate attributes. The random forest algorithm is selected to resemble the decision tree. The tree is trained by using a randomly chosen subset of attributes. This process overcomes the overfitting caused by training the model. Voting is cast through a customized voting system for decision making. Mobile applications’ contexts for microservices are compared through these comparable solutions. All three algorithms are used to compare the results. Based on selecting the appropriate algorithm, NB is a faster algorithm. NB is proficient in describing the dependencies among presented attributes. In NB, portraying the knowledge presented through probability is not easy.

However, although C4.5 learns slowly, its decision tree training produces a more effective hypothesis. The results generated through C4.5 are human-readable. Random forest is slower than C4.5, but it presents more effective and robust predictions than other algorithms. The C4.5 algorithm, developed by Ross Quinlan, is a popular decision tree algorithm widely used for classification tasks in machine learning. It employs a top-down recursive approach to build a decision tree from a dataset. Based on information gain, the algorithm selects the best attribute at each node to split the dataset into subsets. This process continues recursively until the tree is complete. C4.5 handles discrete and continuous attributes, making it versatile for various datasets. Its pruning mechanism avoids overfitting, enhancing generalization. The algorithm’s background lies in the ID3 algorithm, and it addresses its limitations. C4.5’s justifications stem from its ability to handle noisy data and produce interpretable trees. It has been applied in diverse domains, including medical diagnosis and finance, showcasing its effectiveness across different datasets. A possible complex hypothesis is generated, and overfitting is reduced for effective task offloading. However, the results are difficult to analyze and predict due to the larger number of trees.

Multiple techniques are used in learning and prediction for proposed reinforcement learning. The primary objective is to identify the optimal and most efficient approach for acquiring a model for energy optimization using machine learning methodologies. This entails periodically addressing poor solutions. Based on the results, the authors select ϵ−Greedy, where the agent determines the best actions based on the probability of their effects. 1−ϵ is the most suitable activity probability for the most appropriate model learning. Randomized activities are selected for the likelihood of method selection. Equation (2) is chosen as a tuning parameter. The equation shows a set of two final decision values for ϵ; if 0 is computed for the task, it indicates not to offload the task, and 1 indicates that the task should be offloaded to MCC.

(2) $$\epsilon \in \left\{0,1\right\}$$

In this approach, the probability of randomized selection, i.e., ϵ, is constant, but in reinforcement learning, it diminishes with understanding.

### 3.1. Context-Aware Energy Optimization Using Reinforcement Learning for Mobile Cloud

This passage presents a learning agent for adaptive task scheduling and energy optimization approaches. This agent is characterized by a complete set of parameters, including L, K, C, A, P, TD, T, R, and D. This section provides detailed information on the many elements of this framework, which include the learning module (L), knowledge (K), task context set (C), attribute set (A), processing module (P), training dataset (TD), computational tasks (T), results (R), and decision set (D). The process involves the estimation of costs, the retention of outcomes, and the implementation of a context-aware cost-efficient strategy to enhance corporate value. The system’s universal and adaptive character is emphasized since allocated weights for prosperous application areas determine criteria and priorities. The authors must define the learning agent for adaptive task scheduling and energy optimization techniques in this context. Equation (3) illustrates the learning agent in the system:

(3) $${LA}_{RL}\leftarrow \left(L,K,C,A,P,TD,T,R,D\right)$$

where L represents the learning module; K signifies the knowledge acquired during training; and C defines the task context set, including elements such as connection, battery, data, time, related data, and task state. A is the collection of characteristics specified for tasks and their outcomes on mobile devices. P represents the processing module, TD represents the training dataset, T describes the computing tasks performed on mobile devices, R describes the results acquired from task execution, and D represents the decision set after the probability analysis. In Equation (3), D can be written as in Equation (4):

(4) $$D\leftarrow \left\{d_{1},d_{2},d_{3},\ldots \ldots \ldots .,d_{n}\right\}$$

The input data processing module is described in Equation (5). x is the normal module that explains the context.

(5) x←C×T

The pair of values in Equation (5) are described using the pair of values through x, which explains the equation. So, yields of attributes for O∈A are declared in Equation (6):

(6) $$x^{o}=\left(o_{1}\left(x\right),o_{2}\left(x\right),o_{3}\left(x\right),\ldots \ldots \ldots ,o_{n}\left(x\right)\right)$$

Cost prediction is used to retain comprehensive information using variable K. When K displays empty values and all other values are randomized, an efficient and economical method is indicated.

So, d∈D. There are two types of services that the authors use in this context. One is local mobile services, and the second is mobile cloud services. Both services take part in the task of microservices processing. The authors apply decision parameter d to examine such services. The results of modules such as calculation time $C_{time}\left(x,d\right)$ and battery consumption $B_{consume}\left(x,d\right)$ are stored using computational values of P.P is a module that is used to obtain the results of executions, and it is defined through Equation (7), where r∈R:

(7) $${Res}_{total}^{Mobile}\leftarrow \left\{r_{1},r_{2},r_{3},\ldots \ldots \ldots \ldots ,r_{n}\right\}$$

The resulting storage module stores the results obtained through training data and generated knowledge, as shown in Figure 1. The training data (${TD}_{results}$) are stored using $x^{o}$ and d as decision functions. Equation (8) defines a complete example of stored data TD.

(8) $$x^{D\cup A}=\left(\begin{array}{c} o_{1}\left(x\right),\;o_{2}\left(x\right),\;o_{3}\left(x\right),\;\ldots \ldots \ldots ,o_{n}\left(x\right),\;r_{1}\left(x,d\right),\;r_{2}\left(x,d\right),\;r_{3}\left(x,d\right)\\ \ldots \ldots ,r_{m}\left(x,d\right),\;d\; \end{array}\right)$$

Equation (8) defines the complete data stored with values to determine the whole context of the application. This ranges from mobile or cloud computing services to knowledge stores and dataset value predictions. The example demonstrates improvements in a firm’s working capital by optimizing task processing. Discretization is performed when our learning algorithm performs classification as an alternative to regression. Moreover, the optimal values are achieved through provided and effective management programs. Equation (9) shows the predicted expenses when processing the module. P defines the prediction rates for d∈D. $r_{i}\in R_{es}$ defines the prediction values for the processing modules defined under the leadership of other prediction module expenses, such as $P_{expense}\left(d,x\right).$

(9) $$P_{expense}\left(d,x\right)=\sum_{j=1}^{m}{Rw}_{i}*r_{i}(d,x)$$

where ${Rw}_{i}$ represents the weights assigned to the results for $r_{i}.$ The authors establish the criteria or priorities for successful application domains by assigning weights. The findings indicate that the system has a universal and adaptable nature, effectively fulfilling the needs and preferences of users.

Prediction module P helps to predict successful execution, and it makes predictions with the very lowest rates of expenses. Additionally, the P module successfully predicts the execution requirements of the proposed model. This model works on local machines and mobile cloud VMs, so the authors avoid local optima. The e−greedy strategy is applied to achieve local optima. The authors use a suboptimal solution to the problem based on time. The machine learning module executes the suboptimal solution. Algorithm 1 provides a comprehensive explanation of the functioning of probability module P, including all the necessary stages. The machine learning technique defines suboptimal decision making for task scheduling for microservices. The main steps are illustrated in the following points based on Algorithm 1:

- **Step 1:** Determine every task that is to be passed to the machine.
- **Step 2:** For example, t∈OCR, and w∈Wifi.
- **Step 3:** Use machine learning prediction, and train the model.
- **Steps 4 and 5:** Train the model using the predicted technique.
- **Step 6:** Predict the results of the proposed model using the location, time, energy, and cost constraints.
- **Step 7:** d is the best execution location on a cloud VM.
- **Step 8**: Use random numbers to check the testing and training predictions.
- **Step 9:** Execute the OCR task on mobile cloud VM.
- **Step 10:** No prediction model is detected in the first training phase of ML for task scheduling, so the authors decide to locate and use a random position.
- **Step 11:** Time, execution location, tasks, energy, context, and consumption location are stored in the training dataset used by the authors for training and learning.
- **Step 12:** Apply the learned energy and time prediction models.
- **Step 13:** Go to step 1 for the re-learning and training of the prediction model for the subsequent parameters.

**Algorithm 1:** Proposed ML-Based Adaptation Algorithm **Input:** L is the learning module, K is knowledge learned from training, C is task context set such as connection, battery, data, time, associated data, task state, etc., A
is attribute set defined for tasks and their results from mobile devices, P is processing module, TD is training dataset, T is mobile devices computational tasks, R is followed for results obtained from task execution, and D is decision set after probability analysis. T is declared as tasks, W is Wi-Fi. **Output:** Adoptive Decision to of fload tasks **Steps** Get Task←T get context for task T:x=(t,w)     where t,w∈T×W $if\;\left(K\neq \emptyset \right)\;then$          train_model(K,P,T,A,C,L); $while\left(d\in D\&\&r_{i}\in R_{es}\right)do$ $Predict_{Results\left(\right)}\leftarrow r_{i}\left(d,x\right)$ end while Decision Selection:        $d=argument\left(\min\right)\sum_{j=1}^{m}w_{j}*r_{i}(d,x)\;\;\;{rnd}_{number}\left(\right)\leftarrow \left\{\begin{array}{c} \;num\;\in 0\\ num\;\in 1 \end{array}\right.\;\;\;\;\;if({rnd}_{number}<\epsilon )\;\;\;\;d\leftarrow random\_decision()\;\;\;\;\;\;\;end\;if\;\;\;\;\;\;\;end\;if$ else      d←random_decision(prediction rates)      end else Decision if finalized as d:        $execute\left(task\right)\leftarrow dection(d)$ Train Prediction Model: Storage: $x^{D\cup A}=\left(o_{1}\left(x\right),o_{2}\left(x\right),o_{3}\left(x\right),\ldots \ldots \ldots ,o_{n}\left(x\right),r_{1}\left(x,d\right),r_{2}\left(x,d\right),r_{3}\left(x,d\right),\ldots \ldots \ldots \ldots ,\;\;\;\;\;r_{m}\left(x,d\right),d\right)\in TD$        $\;\;\;\;if\left(time\in learning\right)\;then\;\;\;Knowledge\;learned\;from\;Training\;Data\;(TD)\;\;\;\;\;\;K\leftarrow L_{TD}\;\;\;\;\;\;\;End\;if$ Go to Step 1

### 3.2. MCVS Scheduling to Offload Tasks

Task scheduling under the machine learning techniques provides the results under the constructions of the different methods. Algorithm 2 shows the Mobile Cloud Virtual Server (MCVS) task scheduling algorithm with context-aware resource utilization. The algorithm takes the input for the heterogeneous microservices tasks from the machine learning servers to promote MCVS task scheduling.
**Algorithm 2:** MCVS Scheduling Algorithm **Input:** Input data from Table 1 and Algorithm 1. **Output:** TaskScheduling Steps $T_{i\ldots ..n}\leftarrow \mathrm{Data}(\mathrm{decision});$ : Algorithm 1 Decision function $\mathrm{Init}\;\leftarrow V_{l\ldots n};$ $\rho_{k}\leftarrow \mathrm{Nil};$      : MCVS server with zero attributes. ${\mathrm{Data}\mathrm{attribute}\to D}_{A_{n}};$ $\vartheta_{T}=\sum_{l=1}^{n}L_{j}*R_{j}$   : Cost function of the system ${MCVS}_{k}=\{\mathrm{dataset}\};$ : MCVS server set with data. Start:                 while(H)do While (R) do $\mathrm{While}\;(L_{j})do$ $\mathrm{if}\;((L_{j}\leftarrow R)\neq \emptyset )$ then $\mathrm{Attribute}\;\mathrm{Match}\;\to A_{i..j}$: Match all the attributes with resources. $T_{j}\leftarrow R_{k}$ $d_{r}^{k}\equiv T_{j}$                $T_{j}\left(\mathrm{threshold}\right)\to M_{k}\;\;W_{j}\leftarrow W_{j}\left|\left\{T_{j}\right\}\right|$ $\mathrm{Decision}\left\{H_{\mathrm{kl}},\tau_{k}\right\}=1$                 $K_{j}\leftarrow K_{j}\cup \left\{R_{k}\right\}$ $V_{l\ldots n}\leftarrow U_{\mathrm{jk}}$    : Checking the context of tasks. $K_{i}=K_{i}\cup \left\{K_{i}\right\}$                 $\left\{K_{\mathrm{gi}},K_{g2}\right\}\leftarrow W_{i,j,k};$ $\mathrm{if}({(K}_{g1}>K_{g2})\leftarrow A)\mathrm{then}$ $\mathrm{switch}(T_{i}(K_{g1})\mathrm{to}T_{i}{(K}_{g2}))$ allocate$\left(D_{i(g1)}=One(1)\right)$ ${(V}_{l\ldots n})*\leftarrow E_{\mathrm{ij}}$         : Optimal Assignment             $else\;if\left(T_{i}\leftarrow K_{g2}=Null\right)then\;\;\;\;\;M\leftarrow M\left|K_{g2}\right|\;\;\;\;K_{g2}(Limited\;cost\;E)\;\;\;\;\;while\;End\;\;\;\;\;While\;End\;\;\;\;\;Loops-End$

### 3.3. Complexities of Algorithms

**Figure 2:**
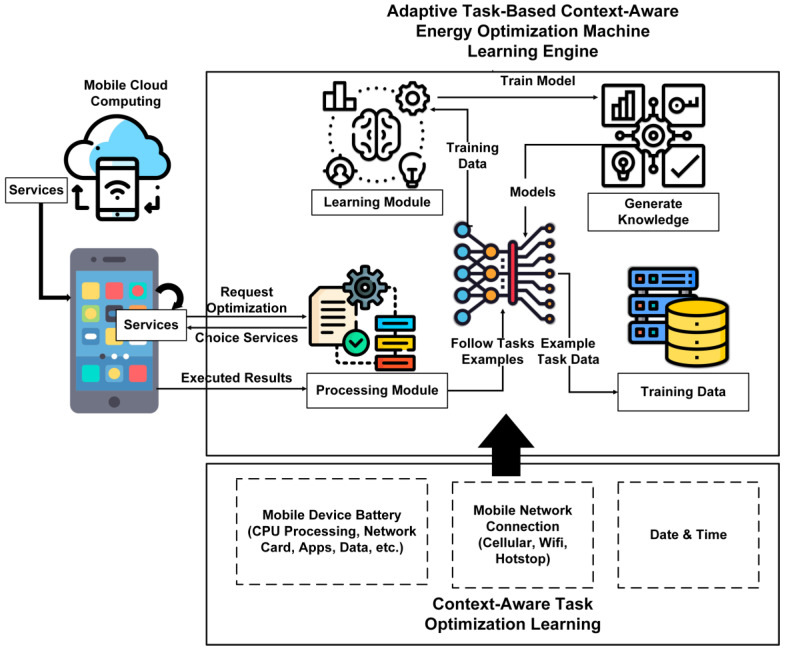
The architecture of adaptive machine learning mobile cloud task scheduling system.

Algorithm 1, a machine learning decision-making algorithm, contains the resources corresponding to the training and testing data listed in the correspondence between the research results. The authors train and test machine learning models with task scheduling algorithms with the aim of using a mobile cloud for microservices-based tasks. In Algorithm 1, the authors take the data as a source with the decision-making attributes to train the model for task scheduling. So, O (log n) indicates the complexity of Algorithm 1. Furthermore, we, the writers, undertake the tasks learned using the machine learning algorithm. Next, we input them into the decision-making server to complete the transition to mobile-cloud-based virtual machines. The complexity of Algorithm 2 is O (n log n), which is based on the computation of task scheduling. Figure 2 shows the proposed architecture with an implementation scenario.

### 3.4. Task Scheduling for Single-Objective and Multi-Objective Perspectives

This study focuses on the crucial problem of context-aware task scheduling in mobile cloud orchestration for microservices application and cost optimization. Regarding cost-effectiveness and resource allocation, task scheduling is essential to meet the goals of both users and suppliers. This study suggests expanding the task scheduling strategy to include single- and multi-objective viewpoints in order to strengthen it. The task scheduling method can efficiently allocate resources based on contextual information. This is accomplished by considering many goals, such as optimizing response time, optimizing resource utilization, and reducing energy use. The system acquires knowledge from historical data and makes astute assessments using machine learning techniques. This optimization minimizes the overall expenses of operating microservices applications inside a mobile cloud environment. This addition offers a more thorough and all-encompassing task scheduling method, enhancing the proposed resource allocation framework’s general efficacy and efficiency.

Task scheduling is crucial to mobile cloud orchestration since it dramatically influences users’ and providers’ goals. The task scheduling algorithm may allocate resources more intelligently by considering numerous objectives, such as reaction time, resource utilization, and energy usage. This multi-objective approach ensures that the system maximizes cost efficiency while considering different performance metrics. Additionally, it facilitates a comprehensive evaluation of various compromises. The task scheduling algorithm may also learn from past data, adapt to dynamic situations, and make data-driven judgments when machine learning is incorporated. The proposed resource allocation approach aims to achieve cost optimization for microservices applications in mobile cloud orchestration. The results are obtained using context-aware tasks and machine learning algorithms. Expanding this paper’s focus to task scheduling improves the research’s overall efficacy and efficiency by addressing a crucial component to achieve defined objectives.

## 4. Performance Evaluation

This paper describes the implementation of experiments using developed software for testing various parameters related to the cloud. Two main parameters, optimization and testing, are emphasized, measuring time, power consumption, and cost estimation. The system implementation involves designing mobile applications for interaction with mobile clouds using Android Studio and a Huawei Y9 emulator. The components include a Mobile Application User Layer, a Mobile Cloud Layer for resource management, and a Mobile Agent Layer for task offloading through a machine learning engine. The machine learning algorithm is implemented using Python, evaluating and classifying various algorithms. A dataset is generated randomly, and parameters like the total length, the number of tasks, and CPU utilization are computed for 20,000 records in the dataset. Evaluation metrics such as precision, recall, and F1 score are employed for machine learning performance and mean squared error with the mean absolute error.

Cohen’s kappa is used for assessing the task scheduling algorithm. Experiments are performed through developed software that tests the possible response to the listed tests. All the results are generated and provided by considering the possible solution. The developed application works on two main parameters: one works on optimization parameters, and the second performs several tests. Both techniques use the provision and number of parameters for development. The tests are conducted with multiple parameters. The methodology measures the time power consumed by the system and estimates cost with individual results. Classification is performed to test the number of parameters in the series. The cost is computed when tasks are stuck in error conditions, an effective mechanism to support analyses.

### 4.1. System Implementation

**Figure 3:**
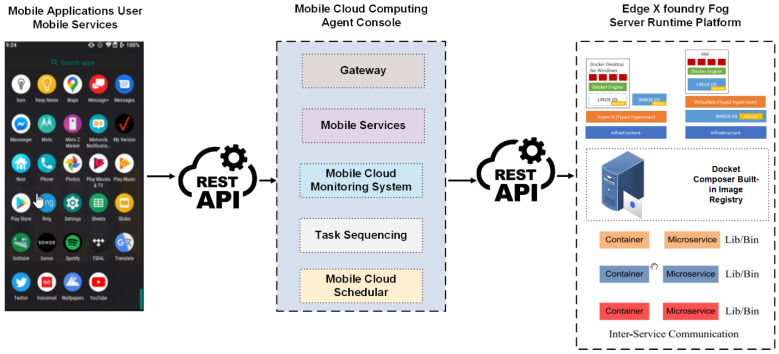
Mobile application design architecture for proposed approach.

For system implementation, the authors design a mobile application that can interact with a mobile cloud using Mobile App Developer IDE [36]. The app is developed using Android Studio over Huawei Y9 with an emulator [37]. Figure 3 shows the implementation details, with a description of the design of the actual mobile application. In this section, the authors work on three main components for implementation, i.e., the Mobile Application User Layer, the Mobile Cloud Layer to manage resources, and the Mobile Agent Layer to offload the selected tasks through the machine learning engine [38]. The authors design all these through the Agent Console REST Application Programming Interface (API) [39].

The JavaScript Object Notation (JSON) library implements the request–response from the user to the mobile cloud and vice versa using the Gateway Interface. Using JSON, the console interface reads the implemented API requests. The Request Optimizer is also implemented to select the tasks requested for the processing module to execute in MCC. The responsibility of the Request Optimizer is to check the listing of tasks and system stability during offloading. Java Runtime Virtual Machine (JVM) runs the Java programs in the implementation section to offload the tasks. All the microservices are designed using container-based implementations in IDE. JVM works like Docker V.M. Every container is registered with the MCC server using registry services. The consumption is suitable for offloading tasks. REST API helps achieve the end-time VM-based implementation of inter-service microservices [10].

The machine learning algorithm is implemented using Python in PyCharm with customized coding. The evaluation and classification are handled through Python in PyCharm for Naïve Bayes, Simple Logistics, Decision Table, OneR, random forest, JRip, and Sequential Minimal Optimization (SMO) [40]. The processed and sanitized CSV dataset file is managed using the Jupyter Notebook integrated development environment (IDE) with the assistance of the Spark read function. The columns within the dataset are adjusted to their actual values before being loaded into the PySpark.ml machine learning algorithms. The manipulation of the dataset column focuses on handling labeled data models using PySpark. The feature vector technique divides the dataset into separate training and testing sets. In this scenario, the authors allocate 50% of the available dataset to training and the remaining 50% to testing. The dataset is prepared to represent data in a vectorized form, resembling libraries, which enables the PySpark library to construct machine-learning-based models. The dataset is input into machine learning algorithms and converted into the requisite columns. Afterwards, the model is used as input for the training and testing procedures.

### 4.2. Dataset Generation and Description

A dataset is generated using the system implementation simulation scenario through the application. The tasks are created with a random length between 5000 and 8000 MIPS. In each set, the number of tasks is 500–750. The scheduling algorithm is presented in Section 2, passing all sets of tasks. The tasks comprise Makespan, cost management, throughput, energy optimization, task sequencing, the task failure ratio, server optimization, and fault tolerance. The features in the dataset, like the total length, number of tasks, machine numbers, average load, and average arrival time, are computed for the number of VMs. These features make a single record for the dataset. The simulations are repeated 5000 times to produce a complete dataset. The CSV file is extracted at the end of the whole simulation using the abovementioned implementation. Again, the process is repeated with 20,000 iterations to complete it. The main features in the dataset are as follows:

- The total length of each task;
- The number of tasks;
- The number of VMs;
- Average load;
- Task type;
- The CPU time required;
- Difference time in average arrivals;
- CPU utilization;
- Bandwidth utilization;
- Task failure ratio;
- Task boot time.

The primary parameters in a dataset are MCC server utilization, fault tolerance, task deadline completion, task boot time, cost efficiency, resource handling, and task schedule latency. It consists of 20,000 rows of data. Table 2 and Table 3 show the sample dataset. The dataset, generated through 20,000 iterations, exhibits diverse tasks, each comprising Makespan, cost management, throughput, energy optimization, task sequencing, the task failure ratio, server optimization, and fault tolerance. The tasks vary in length (5000–8000 MIPS) and quantity (500–750 per set). Features, such as the total length, number of tasks, machine numbers, average load, and average arrival time, are computed for each set of VM, creating a comprehensive record. With parameters like machine learning, MCC server utilization, and fault tolerance, the dataset represents multifaceted aspects of performance analysis. This rich variety ensures a robust training set for the machine learning model, fostering adaptability to real-world scenarios.

A confusion matrix measures the machine learning performance. The neural network method is evaluated using a confusion matrix. Precision works by using the model’s predictive positive value. This shows the precision or accuracy of the model. Equation (10) is implemented for precision:

(10) $$\mathrm{Precision}=\frac{\mathrm{TP}}{\mathrm{TP}+\mathrm{FP}}$$

Recall shows our model’s actual positive values based on possible labeling. Equation (11) shows the recall implementation:

(11) $$\mathrm{Recall}=\frac{\mathrm{TP}}{\mathrm{TP}+\mathrm{FN}}$$

The F1 score shows the balance values between precision and recall. Equation (12) shows the F1 score implementation:

(12) $$F1\mathrm{Score}=2*\left\{\frac{\mathrm{Precision}*\mathrm{Recall}}{\mathrm{Precision}+\mathrm{Recall}}\right\}$$

Each parameter shows the results obtained from the machine learning implementation. Afterwards, the task scheduling algorithm is trained to schedule the tasks based on these parameters. The mean squared error (MSE) [29] measures the mean squared difference between predicted diabetes cases and actual cases in Equation (13):

(13) $$MSE=\frac{1}{n}\sum_{i=1}^{n}{\left(y_{i}-{\overset{ˇ}{y}}_{i}\right)}^{2}$$

The mean absolute error (MAE) [30] measures the number of errors in classification from Equation (14):

(14) $$MAE=\frac{\sum_{i=1}^{n}\left|y_{i}-x_{i}\right|}{n}$$

Cohen’s kappa [31] measures the performance of the model and is shown using Equation (15):

(15) $$KAPPA=\frac{P_{0}-P_{e}}{1-P_{e}}$$

Table 2 summarizes the classification model performance. The outcomes are compared with different execution estimates with the various evaluation parameters described above.

Based on the confusion metric values in Table 2, Table 3 shows the performance results. The results are compared against each parameter, such as precision, recall, and F1 score.

**Figure 4:**
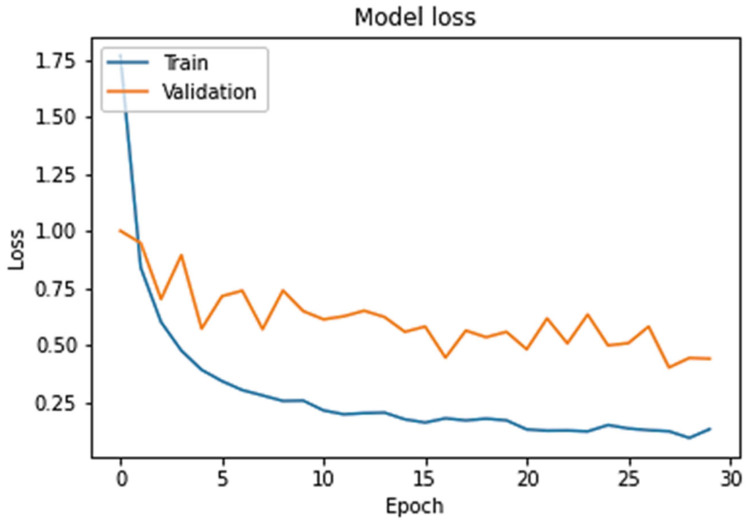
Training of machine learning model.

Figure 4 shows the network’s repeated training success in the validation test. After 30 repetitions, the model loss reduced rapidly. The blue line in the graph shows the loss during training. Repeated training quickly reduced the loss, and the model accuracy promptly increased.

**Figure 5:**
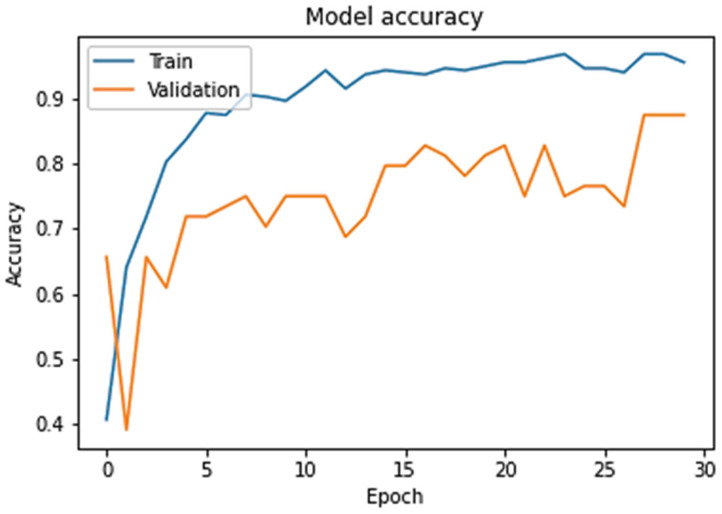
Model accuracy at 30 epochs.

**Figure 6:**
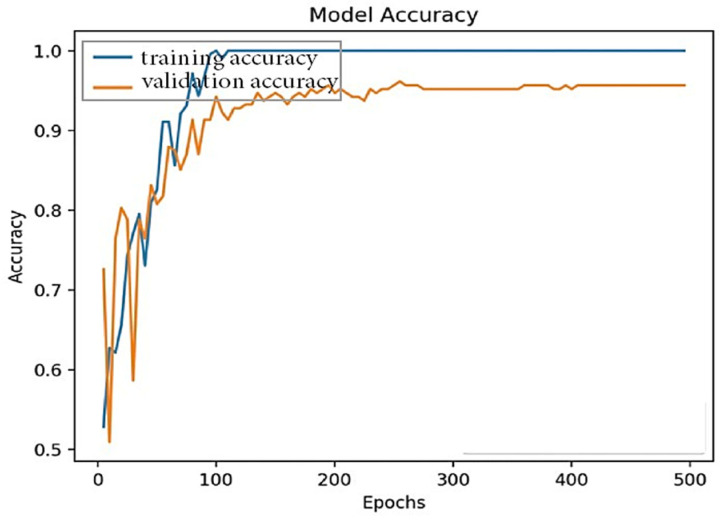
Model accuracy at 500 epochs.

Moreover, a trained model was tested in each class to evaluate the model’s performance. Tests were performed on all the tasks in the verification set. The results show how many attributes in each class were accurately predicted. Figure 5 shows the model accuracy during training and testing. The following graph shows that the model accuracy increased rapidly with an increasing number of iterations. Figure 6 shows the model accuracy at 500 epochs for training and testing the accuracy values.

**Figure 7:**
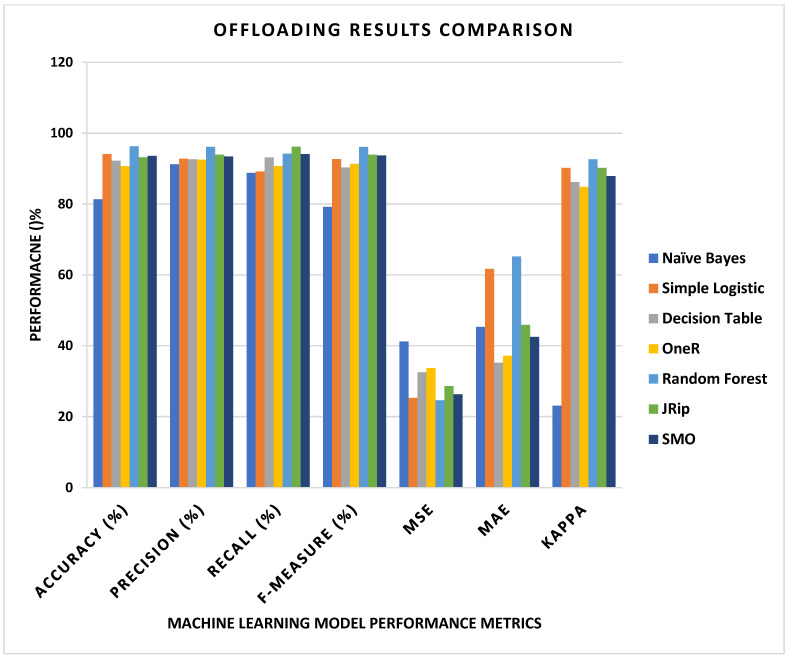
Proposed machine learning model evaluation comparison results.

In the model results, it is notable that the model’s accuracy increases rapidly, and the loss of the model decreases over the maximum iterations (epochs) applied to train it. Figure 5 and Figure 6 show the results of the epoch and accuracy graphs. Figure 7 shows the results of the parameters for the proposed machine learning algorithm, Algorithm 1, and their comparison. Accuracy, precision, recall, F1 score, M.S.E., M.A.E., and KAPPA are the leading performance metrics selected for the comparison. The results are from Table 3, which shows the actual results from different perspectives to present a complete comparison, as shown in the figure.

### 4.3. Comparison of Offloading Framework Results

**Figure 8:**
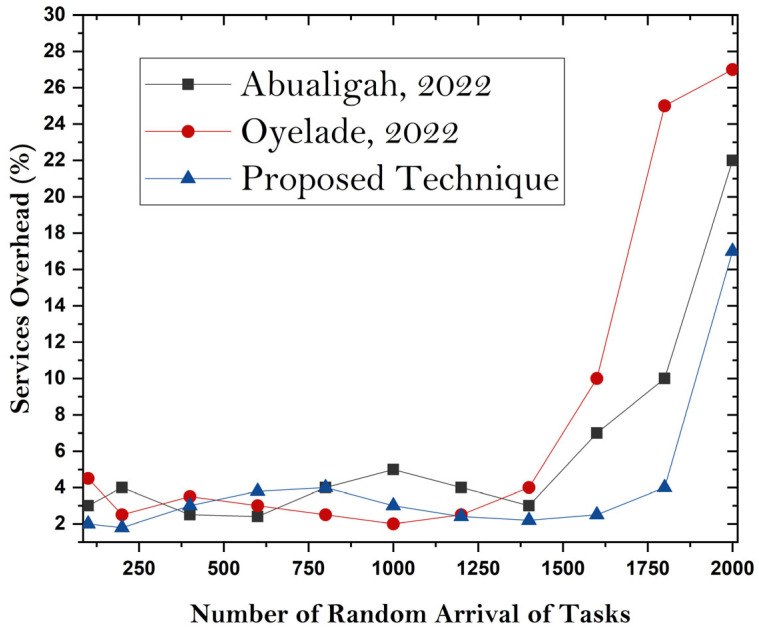
Boot time of microservices [31,32].

**Figure 9:**
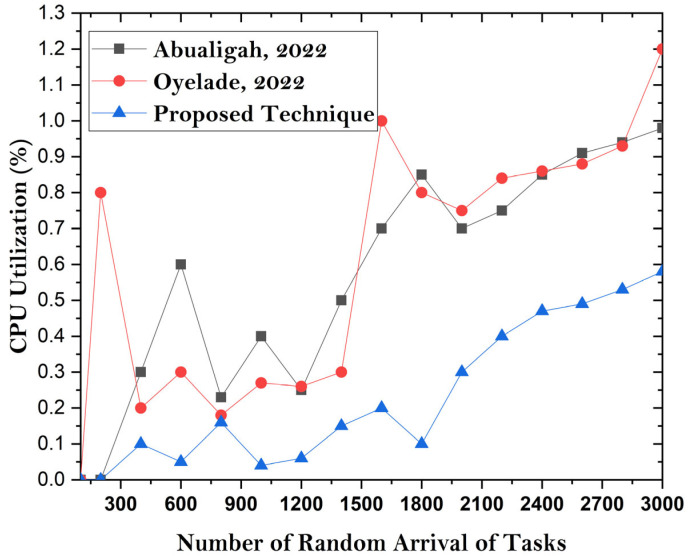
CPU utilization of resources [31,32].

**Figure 10:**
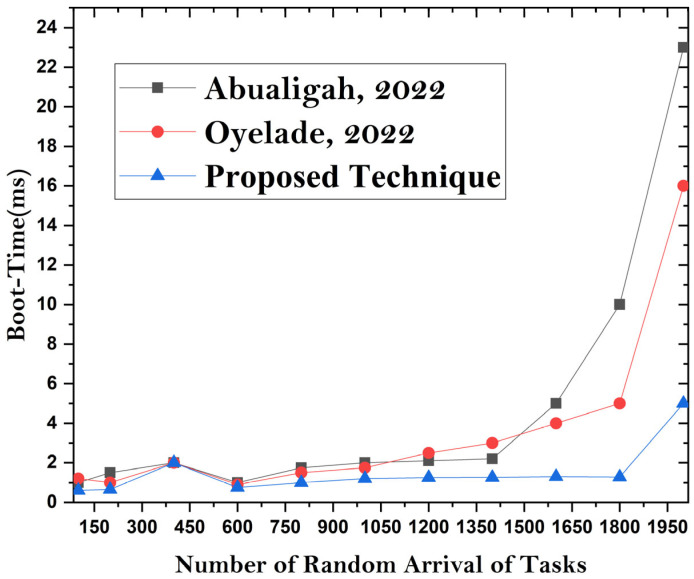
The overhead of microservices [31,32].

This discussion compares the offloading frameworks with existing implementations in terms of the bootup time of microservices-based MC applications. In the proposed undertaking, it is found that the container-based MCC offloading framework has a lower bootup time than existing heavy-computation VM frameworks. Resource utilization is effectively improved in the proposed system compared with that of existing methodologies. Figure 8, Figure 9 and Figure 10 show the simulation results for boot time, CPU resource utilization, and overhead parameters. The figures show that the proposed framework has an improved boot time, overhead time, and resource utilization. The reason for achieving accurate results is the lightweight design of the utilized VM server compared to the heavy and resource-intensive design of mobile devices. So, task processing and resource utilization are effective ways to achieve enhancements.

### 4.4. Task Failure Ratio

**Figure 11:**
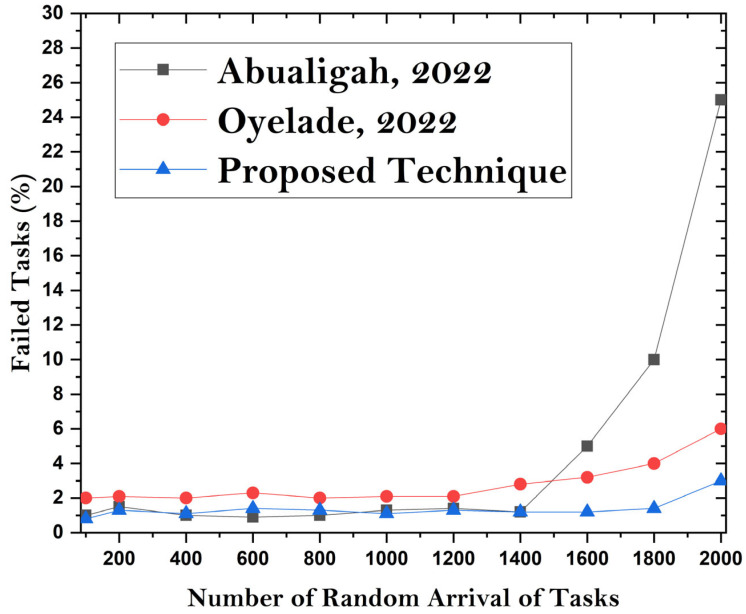
Failure ratio of task scheduling [31,32].

**Figure 12:**
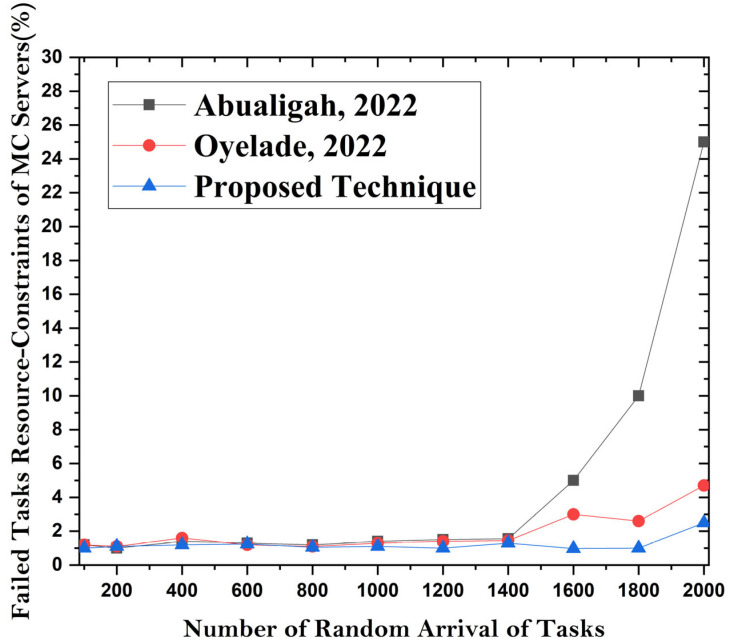
Resource-constrained task failure on mobile cloud servers [31,32].

Task failure is linked to fault tolerance in the proposed methodology. The task failure ratio is used to compare the existing approaches with the predefined implementation of the task processing module. In the existing methods, task scheduling is only applied with little or no handling of fault tolerance using proposed machine learning algorithms. The authors handle the task failure ratio by using machine learning algorithms that effectively improve system performance. Figure 11 elaborates on the handling of fault tolerance using the proposed methodology compared with the existing state-of-the-art techniques. The results in Figure 12 show that the proposed method performs better than the existing methods when using dynamic VM servers for MCC with machine learning algorithms. The proposed task scheduling framework works effectively by using the dynamic allocation of microservices container-based mobile tasks. The resources and mobile environment are effectively managed and handled through the proposed approach. The resources are effectively balanced with minimum or no fault tolerance. All the deadlines for the resource-constrained tasks are met under low- and high-level system utilization. The execution cost is handled by directing cost-efficient and resource-effective tasks to mobile cloud applications.

### 4.5. Response Time

**Figure 13:**
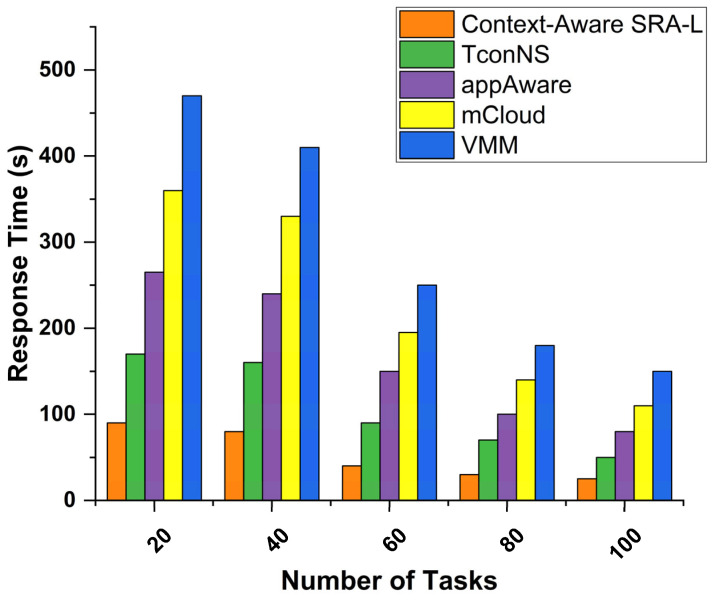
Comparison of response time.

Figure 13 compares the response time with context-aware task offloading in mobile devices. The figure shows a comparison of the response time between the proposed Context-Aware Smart Resource Allocation-Learning (SRA-L) with the TConNS, appAware, mCloud, and VMM [41] algorithms when processing different numbers of tasks (*n* = 20, 40, 60, 80, 100). The average response time of Context-Aware SRA-L is about 29% lower than that of the other schemes.

## 5. Conclusions, Research Contribution, Limitations, and Future Work

Multiple studies have been carried out to reduce the power consumed by mobile devices by improving cost estimations. This study found that no solution currently applies machine learning to microservices for work offloading and scheduling. The proposed technique minimizes the expenses associated with mobile devices and reduces power usage. The proposed model successfully and effectively enhances mobile devices’ decision-making power, thus overcoming these recent challenges. In select problems, the machine learning algorithm allows for the optimization of and a reduction in the service execution time of mobile devices. This solution is adopted mobile application-based microservices like face recognition, continuous weather information, and a3D online and offline game scenario. The authors implement the solution using Android OS with the AWS cloud framework. The experimental findings demonstrate that the suggested machine learning approach significantly decreases power usage.

Furthermore, it reduces the expenses associated with application offloading. In addition, we, the authors, show that the power consumption of mobile devices throughout the learning process is minimal. This is a clear indication of the importance of our suggested approach. This research demonstrates that our proposed solution’s decision-making system effectively enhances and implements task scheduling in the mobile cloud computing environment. As a result, mobile devices see a significant decrease in power consumption and service execution time. Using data in the learning process significantly improves the operational principles, thus optimizing the system’s efficiency. These models are adopted to strengthen the working of the global knowledge discovery area and provide an effective monitoring and control system.

### 5.1. Research Contributions

This work presents a new method for context-aware task offloading in mobile devices, with the aim of preserving scarce resources in mobile apps. Our approach uses a microservices architecture that incorporates a context-aware machine learning model. This model is based on supervised machine learning and aims to optimize cost and energy efficiency while enhancing system performance. In addition, the authors use a reinforcement learning-based method and a tailored task scheduling algorithm, considering fault tolerance and resource limitations, to achieve improved task offloading efficiency. The research includes the use of server-based optimized work scheduling strategies, which apply a service matching technique that relies on machine learning characteristics. The graphical results illustrate the effectiveness of the suggested model, showing significant enhancements in the service offloading rate and CPU use compared to existing methodologies, especially in the field of mobile cloud computing (MCC). In addition, our microservices-based task scheduler, which utilizes machine learning, effectively tackles the issues of optimizing computational cost and energy consumption related to work management on mobile devices, yielding efficient task scheduling outcomes.

### 5.2. Limitations

The main limitations of the proposed work are as follows:

- This research is limited to microservices-based context-aware tasks and does not apply to the other normal functions that flow through MCC on mobile devices.
- This research is limited to MCC application processes and cannot handle the other tasks on mobile devices.

### 5.3. Future Work

The authors plan to implement microservices-based Internet of Medical Things applications in the future, as medicine is an evolving field with many IoT-enabled patient and paramedical investigations. Both transient failure and security are considered during microservices task offloading frameworks. Besides the plan, this research is limited to context-aware tasks with microservices-based development for mobile cloud devices. Another limitation is that the tasks are homogeneous.

## Figures and Tables

**Table 2:** Comparison with related work based on characteristics of proposed system.

Algorithms	Accuracy (%)	Precision (%)	Recall (%)	F-Measure (%)	MSE	MAE	KAPPA
Naïve Bayes	81.3	91.2	88.8	79.2	41.2	45.3	23.1
Simple Logistic	94.1	92.8	89.2	92.7	25.3	61.7	90.2
Decision Table	92.2	92.6	93.1	90.3	32.5	35.2	86.2
OneR	90.7	92.5	90.7	91.3	33.7	37.2	84.8
Random Forest	96.3	96.1	94.2	96.1	24.6	65.2	92.6
JRip	93.2	93.9	96.2	93.9	28.6	45.9	90.2
SMO	93.6	93.4	94.1	93.7	26.3	42.5	87.9

**Table 3:** Comparison of performance evaluation metrics.

Performance Measure				
Parameter	Precision	Recall	F1 Score	Accuracy
Throughput	0.91	0.87	0.97	0.95
Makespan	0.90	1.00	0.95	0.97
Cost	0.89	0.98	0.95	0.975
CPU Utilization	0.98	0.99	0.96	0.98
Fault Rate	0.97	1.00	0.99	0.985

## Data Availability

Due to the ongoing nature of the project on which this research is based, the original data cannot be publicly shared. For those interested in this study, please contact the corresponding author of this paper for further information and access to the relevant data.
